# Should We Pay More Attention to Left Atrial Appendage Physiology During Rhythm Restoration?

**DOI:** 10.1016/j.jaccas.2026.110540

**Published:** 2026-10-28

**Authors:** Daisuke Ueshima

**Affiliations:** Department of Cardiology, Kameda Medical Center, Kamogawa, Chiba, Japan

**Keywords:** atrial fibrillation, echocardiography, thrombus

**Figure:**
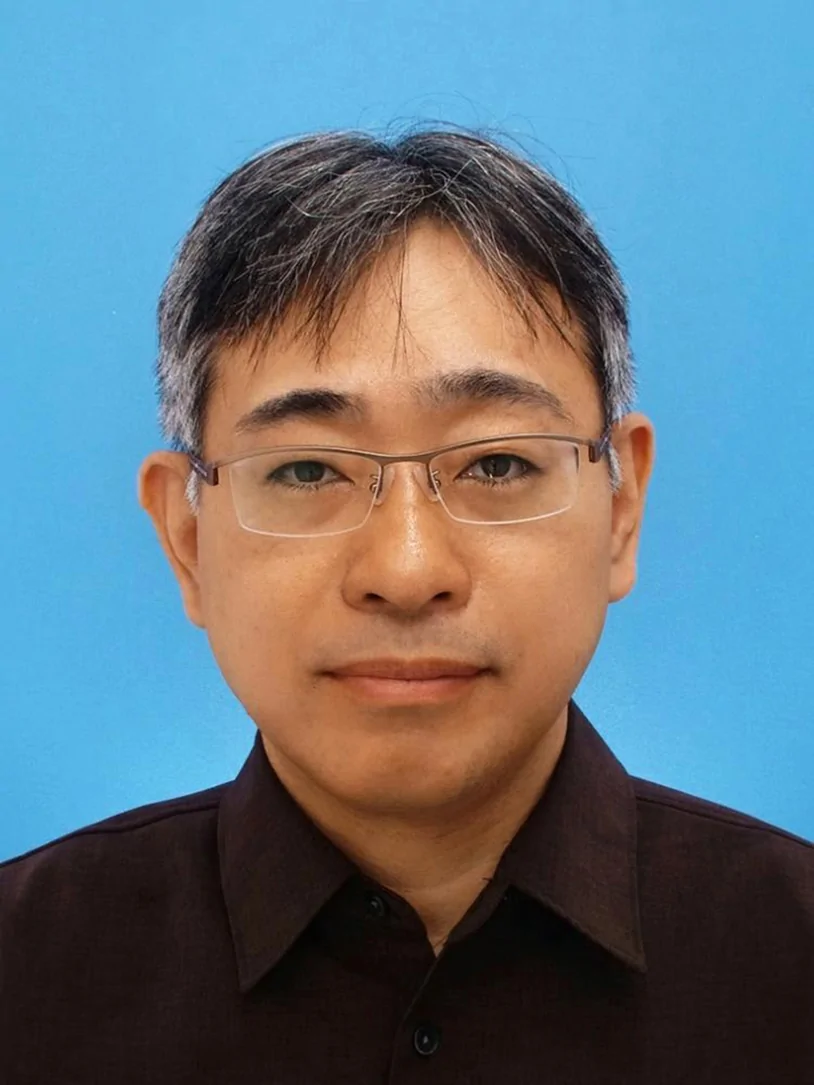

Left atrial appendage closure (LAAC) has become an established treatment for stroke prevention in patients with atrial fibrillation (AF) who are unsuitable for long-term oral anticoagulation. Catheter ablation is also an established rhythm-control strategy for selected patients with AF.^1^^,^^2^ As the indications for both procedures continue to expand, an increasing number of patients become candidates for both LAAC and AF ablation. Accordingly, a combined procedure has become an attractive strategy because it avoids repeated transseptal puncture, shortens the overall treatment course, and may reduce health care costs. Accumulating experience suggests that combined LAAC and AF ablation can be performed safely in selected patients^3^; however, important questions remain regarding how these 2 procedures should be combined.

One of the most important unresolved questions is the optimal procedural sequence for combined LAAC and AF ablation. The COMBINATION (Strategy Optimization for the Combined Procedure of Left Atrial Appendage Occlusion and Catheter Ablation in Patients With Atrial Fibrillation) randomized trial demonstrated that an occlusion-first strategy was associated with superior event-free survival for the primary composite endpoint, including stroke or transient ischemic attack, device-related thrombus, clinically relevant bleeding, and cardiovascular rehospitalization or death. In addition, occlusion-first sequencing was associated with lower rates of chronic peri-device leak and device-related thrombus during follow-up. Importantly, restoration of sinus rhythm, either by catheter ablation or electrical cardioversion, was incorporated into the procedural strategy, underscoring that cardioversion is frequently performed during combined procedures.^4^ However, thromboembolic protection represents only 1 aspect of procedural success. From the electrophysiology perspective, procedural sequencing also may influence rhythm-control outcomes. Recent observational data presented at the American College of Cardiology Scientific Session suggested that ablation-first sequencing may be associated with better long-term rhythm outcomes.^5^ Although these findings require further confirmation, they suggest that thromboembolic protection and rhythm-control efficacy may not necessarily favor the same procedural sequence. Consequently, the optimal procedural strategy remains uncertain. Many operators may still hesitate to perform cardioversion immediately before or after LAAC, not because of established evidence of harm, but because the immediate mechanical response of the left atrial appendage (LAA) remains poorly understood. The present report provides an important physiological perspective on this unresolved clinical question.

In this issue of *JACC: Case Reports*, Ivanov et al^6^ describe a case series of acute LAA stasis occurring within minutes of electrical cardioversion during LAAC procedures. An ultrasound-enhancing agent was used to distinguish thrombus from incomplete contrast filling. More importantly, restoration of sinus rhythm did not result in immediate recovery of LAA mechanical function. This observation may have important implications for combined LAAC and AF ablation procedures.

Transient impairment of LAA mechanical function after cardioversion has long been recognized.^7^ Reduced LAA emptying velocity, spontaneous echo contrast, and sludge may persist despite successful restoration of sinus rhythm, reflecting delayed recovery of LAA mechanical function. The present case suggests that these changes may develop within minutes after cardioversion and become detectable by transesophageal echocardiography. Whether transient blood stasis progresses to thrombus formation remains unknown; however, this observation suggests that cardioversion may create a transient period of impaired LAA flow immediately after restoration of sinus rhythm. The recovery of LAA function is likely a dynamic process and may vary substantially among patients. AF duration, the underlying atrial rhythm before cardioversion, the extent of atrial remodeling, and the amount of atrial injury related to ablation may all influence the mechanical recovery of the LAA. The relative contribution of each of these factors remains unknown. This variability may explain why transient LAA dysfunction becomes clinically apparent in some patients but not in others.

The present case may also offer a physiological explanation for the clinical observations reported in the COMBINATION trial. In that study, occlusion-first sequencing was associated with fewer thromboembolic events than ablation-first sequencing during combined procedures.^4^ One possible explanation is that cardioversion performed during rhythm restoration may induce transient LAA dysfunction before complete mechanical recovery, creating a period of impaired appendage flow. If transient LAA flow impairment contributes to thrombus formation, exclusion of the appendage before rhythm restoration could theoretically reduce this risk. However, this interpretation remains speculative. Although the present case cannot establish causality, it illustrates how carefully documented physiological observations from individual cases may help interpret findings from larger clinical studies. Such observations complement randomized evidence by providing a biologically plausible basis for clinical observations. The present case does not establish a causal relationship between transient LAA flow impairment and thromboembolic events, nor does it support one procedural sequence over the other. Rather, it adds an important mechanistic perspective to the ongoing discussion regarding procedural sequencing.

An important unanswered question is whether marked LAA flow impairment immediately after cardioversion should influence intraprocedural decision-making. The present report cannot answer this question, but it clearly demonstrates that significant impairment of LAA flow can develop within minutes after restoration of sinus rhythm. If marked LAA flow impairment is observed immediately after rhythm restoration during a combined procedure, should LAAC proceed without delay, or should the procedural strategy be modified? At present, no evidence supports either approach. Similarly, whether transient LAA flow impairment contributes to thrombus formation, device-related thrombosis, or clinical thromboembolic events remains unknown. These questions should be addressed before procedural strategies are modified.

The present report does not resolve the debate regarding combined LAAC and AF ablation or determine the optimal procedural sequence. Instead, it provides an important physiological insight that may help explain why procedural sequencing matters. As combined LAAC and AF ablation continue to evolve, a better understanding of LAA physiology during rhythm restoration may become increasingly important for optimizing procedural strategies.

## Funding Support and Author Disclosures

The author has reported that he has no relationships relevant to the contents of this paper to disclose.
